# Models for Oral Biology Research 2.0

**DOI:** 10.3390/biomedicines12122804

**Published:** 2024-12-10

**Authors:** Fernando Capela e Silva, Elsa Lamy, Paula Midori Castelo

**Affiliations:** 1 Department of Medical and Health Sciences, School of Health and Human Development, Colégio Luís Verney, University of Évora, 7000-671 Évora, Portugal; 2 MED—Mediterranean Institute for Agriculture, Environment and Development & CHANGE—Global Change and Sustainability Institute, University of Évora, 7006-554 Évora, Portugal; ecsl@uevora.pt; 3 Department of Pharmaceutical Sciences, Institute of Environmental, Chemical and Pharmaceutical Sciences, Universidade Federal de São Paulo (UNIFESP), Diadema 09913-030, Brazil; paula.castelo@unifesp.br

The oral cavity constitutes a unique and complex system and environment. It includes the lips, cheeks, teeth, and gums; the soft and hard palates; the floor of the mouth and the tongue; as well as non-visible structures such as muscles, nerves, blood vessels, glands, joints, and the jaws [1] that provide support for vital functions, namely, feeding, breathing, somatosensation, speech, sucking, and swallowing [2]. The oral cavity is constantly exposed to the physical stimuli of chewing on different types of food with different texture, compositions, and temperatures. Biochemical changes also occur inside the mouth due to variations in diet—especially sugar content—and the activity of microorganisms, either commensal or pathogenic ones [1,3]. Diseases originating in the oral cavity can have systemic effects, and systemic diseases can be reflected on and can affect the oral cavity. The first signs and symptoms of many systemic diseases may appear in the mouth [2,4,5].

The study of oral biology and the related tissues, structures, and secretions of the oral cavity involves several disciplines, namely, cellular and molecular biology, biochemistry, biophysics, genetics, microbiology, immunology, and physiology, among others, and requires the development of new models and approaches to understand, prevent, manage, or overcome various human oral or systemic diseases. In this Special Issue, following a first edition on the same theme (Models for Oral Biology Research), we intended to bring together, within the scope of new models for studying oral biology, studies that include new methodologies and perspectives for understanding the biology of oral and systemic diseases and their relative processes. In the end, we compiled eight articles (listed below) that cover important areas of oral biology within an appropriate theoretical framework.

Oral health includes soft and hard structures and tissues that allow people to perform essential functions such as eating, swallowing, breathing, and speaking. Oral health changes throughout the lifespan and encompasses psychosocial dimensions such as self-esteem, well-being, and the ability to socialize and work without pain, discomfort, and constraints, thus being closely related to the individual’s quality of life. According to the World Health Organization (WHO), oral diseases constitute a public health problem in many countries. According to the 2022 WHO Global Oral Health Status Report, oral diseases affect around 3.5 billion people worldwide, with 3 out of 4 affected people living in middle-income countries [2]. The prevalence of oral diseases—mainly dental caries and periodontal disease—continues to increase globally due to the increasing urbanization, social inequalities and educational disadvantages, inadequate exposure to fluoride in water and toothpastes, limited access to oral health assistance, and food insecurity that leads to increased access to foods with high sugar content [2].

Periodontitis and dental caries are the most common diseases of the oral cavity; globally, it is estimated that 2 billion people suffer from caries in the permanent teeth and 514 million children suffer from cavities in the primary teeth [2]. Their main etiological factors are biofilm accumulation and a diet rich in sugar, respectively, and they can lead to pain, impaired mastication due to tooth loss, and an important impact on the quality of life [6,7].

Due to their influence on local and systemic inflammation, oxidative stress and microbiome composition, air pollution, although poorly explored, may represent a modifiable and preventable risk for periodontitis. Marruganti et al. (Contribution 1) investigated the epidemiological association between outdoor air pollution and conditions [particulate matter of 10 μm, ozone, sulfur dioxide (SO_2_), nitrogen dioxide (NO_2_), and humidity] and periodontitis in a representative sample of 42,020 participants from South Korean. Periodontitis was defined according to the Community Periodontal Index (CPI ≥ 3), and the authors applied simple and multiple regression analyses to propose four different models. Some of the parameters studied were associated with periodontitis prevalence, such as particulate matter of 10 μm, ozone levels, humidity, and NO2 levels. The results indicate that several air pollutants were associated with periodontitis occurrence, suggesting that air pollution may be a new modifiable risk factor for periodontitis.

Neopterin, a pyrazino–pyrimidine compound belonging to the pteridine group, is known to be a biomarker associated with cell-mediated immunity, and its levels reflect the stage of activation of the cellular immune system [8,9]. Produced by human monocytes/macrophages and dendritic cells, it is a very important clinical parameter, although its physiological role is not yet clearly understood [9]. Heneberk et al. (Contribution 2) published a review whose purpose was to summarize neopterin metabolism, its detection methods, and its role in inflammatory diseases—mainly periodontal diseases. In addition to a wide range of diseases and conditions in which neopterin levels are altered, in individuals with periodontitis, their levels in oral fluid and gingival crevicular fluid were also increased, suggesting the role of activated macrophages and cellular immunity in periodontal diseases. According to their findings, the assessment of neopterin levels in individuals with periodontitis can be successfully performed in gingival crevicular fluid and oral fluid.

The alveolar bone, the part of the jawbone that holds the teeth and supports oral functions, has a unique metabolism behavior due to the proximity of dental biofilm [10,11]. Sustained chronic periodontal inflammation disrupts the balance of osteoclast–osteoblast interactions, which may ultimately lead to irreversible destruction of the periodontium (i.e., the alveolar bone and periodontal ligament) [10,11]. The review of Tsuchida and Nakayama (Contribution 3) addresses several aspects of the alveolar bone, namely, alveolar bone resorption, periodontal treatment modalities, new classification of periodontal disease, tissue engineering for periodontal tissue regeneration, guided bone regeneration, clinical applications of membranes, implant therapy, bone grafting, and new findings from clinical and basic research that seek to overcome tooth loss and periodontitis. The content of this review addresses an important topic given the high prevalence of periodontal diseases throughout the world and the clinical significance of maintaining a healthy oral environment to provide both direct and indirect positive effects on overall health.

Dental caries can be clinically detected by means of visual–tactile examination of superficial changes in the enamel and dentin due to surface demineralization. Other non-invasive techniques for detecting early tooth decay have been proposed with good results, namely, magnifying loupes, transillumination, light and laser fluorescence and autofluorescence, electric current/impedance, tomographic imaging, and image processing [12,13]. The study of Govind et al. (Contribution 4) aimed to evaluate the effectiveness of a bioactive caries detection dye for the diagnosis and mechanical removal of occlusal and proximal carious lesions. Patients with carious lesions were treated with a rotary technique and dye solution to detect caries and were re-evaluated after 3, 6, and 12 months, applying the FDI criteria. The results indicated that the caries detection dye was successful in helping to identify carious lesions, being effective in removing demineralized tissue with less pain or sensitivity.

Oral cancer is considered an important public health problem, representing the 11th most common carcinoma worldwide [14]. Approximately 90% of total oral malignant conditions are represented by squamous cell carcinomas, probably due to a wide range of risk factors, including, but not limited to, periodontitis [15], alcohol intake, smoking and chewing tobacco, betel quid, and areca nut [14]. The areca nut, also called the betel nut, is the *Areca catechu* seed that grows throughout the tropical Pacific, Asia, and parts of East Africa. It is often chewed wrapped inside betel leaves (paan) or with tobacco (betel quid), and it is an addictive substance consumed by people of all age groups [16]. It has many effects on the human body, affecting almost all organs, with potential carcinogenic activity in the pharynx, esophagus, liver, uterus, and, very particularly, the oral cavity [16]. Areca nut chewing is one of the main risk factors for oral cancer, with a large magnitude of risks reported in studies comparing betel quid chewers and never-users [17]. It can affect the hard tissues, including the teeth, the periodontium, and the temporomandibular joint, as well as the soft tissues, including the mucosa [18], and the risk of oral cancer increases in a dose–response manner. The precise mechanisms by which it stimulates mucosal cells, producing malignancy, and contributes to the progression of oral cancer are not yet fully understood. Wang et al. (Contribution 5) assessed the effects of arecoline, an important alkaloid component of areca nuts, on the S-G cell line of normal human gingival epithelium through cell viability, reactive oxygen species (ROS) levels, protein expression, cell morphology, and gene expression. The authors concluded that arecoline stimulates the production of IL-1 and mitochondria, resulting in the generation of ROS, thus inducing inflammatory response, inhibiting wound healing, and promoting oral cancer through recurrent ulcers.

The mucosal pellicle is a biological film that protects the oral mucosa and participates in lubrication, moisture, and protection against microbial colonization [19]. The pellicle includes secreted soluble mucins (MUC5B, MUC7), membrane-associated epithelial mucins (MUC1), and, to a lesser degree, carbonic anidrase VI, sIgA, and cystatin [20]. The membrane-anchored MUC1, expressed in the superficial layer of the oral mucosal epithelium, especially on the upper surface of epithelial cells, is a factor that enhances mucous pellicle formation by increasing the binding of salivary MUC5B to oral epithelial cells [21]. Nivet et al. (Contribution 6) developed a new in vitro oral mucosa model to investigate the role of the main structural domains of MUC1 using a TR146 cell line, which does not express MUC1 natively. Cells were stably transfected with genes encoding three isoforms of MUC1 that differ in the structure of the two main extracellular domains. The results suggest the involvement of hydrophobic effects in the interaction between MUC1 and salivary proteins and help us to understand the role and structure of MUC1 in the lubrication of the oral cavity, with potential clinical implications for addressing issues related to oral dryness.

In recent years, the field of artificial intelligence (AI) has progressed remarkably, including in healthcare, with enormous potential to improve patient care and quality of life by assisting the clinical practice [22]. The application of AI in healthcare has two main branches: virtual and physical. The virtual component is represented by machine learning (i.e., deep learning), represented by mathematical algorithms that improve learning through experience; the physical component includes increasingly sophisticated objects, medical devices, and robots that participate in the provision of care [23]. In recent years, AI has also been gaining more attention in dentistry for various purposes, such as identifying normal and abnormal structures, diagnosing diseases, and predicting treatment results [24]. In this Special Issue, two articles are included on this subject. Vishwanathaiah et al. (Contribution 7) conducted a review on the applications of AI models in pediatric dentistry. AI can be successfully used in pediatric dentistry with the purpose of making a more accurate diagnosis and assisting clinicians and pediatric dentists in making clinical decisions, developing preventive strategies, and establishing an appropriate treatment plan. The aim of the systematic review by Khanagar et al. (Contribution 8) was to critically evaluate the available evidence on the use of AI in diagnosing, classifying, and predicting oral cancer using histopathological images. The authors concluded that AI has great potential to provide a higher level of precision and accuracy, helping pathologists to improve their diagnostic results significantly and reducing the likelihood of errors. They also suggested that given these advantages, regulatory authorities and policy makers should simplify the processes for approving and marketing these products for use in clinical settings.

## References

[1] Anandharamakrishnan C., Moses J.A., Priyanka S. (2023). The Human Oral Cavity and Oral Processing of Foods.. Food Digestion and Absorption: Its Role in Food Product Development.

[2] WHO (2022). Global Oral Health Status Report: Towards Universal Health Coverage for Oral Health by 2030.

[3] Sampaio A.A., Souza S.E., Ricomini-Filho A.P., Del Bel Cury A.A., Cavalcanti Y.W., Cury J.A. (2019). Candida albicans Increases Dentine Demineralization Provoked by Streptococcus mutans Biofilm. Caries Res..

[4] Ribeiro A.S.P., Marquezin M.C.S., Pacheco E.R.P., Rasera I., Klein M.I., de Vasconcellos S.P., Landgraf R.G., Okamoto D., Calixto L.A., Castelo P.M.C. (2023). Bypass gastroplasty impacts oral health, salivary inflammatory biomarkers, and microbiota: A controlled study. Clin. Oral Investig..

[5] Botelho J., Mascarenhas P., Viana J., Proença L., Orlandi M., Leira Y., Chambrone L., Mendes J.J., Machado V. (2022). An umbrella review of the evidence linking oral health and systemic noncommunicable diseases. Nat. Commun..

[6] Kwon T., Lamster I.B., Levin L. (2021). Current Concepts in the Management of Periodontitis. Int. Dent. J..

[7] de Albuquerque L.S., de Queiroz R.G., Abanto J., Strazzeri Bönecker M.J., Soares Forte F.D., Sampaio F.C. (2023). Dental Caries, Tooth Loss and Quality of Life of Individuals Exposed to Social Risk Factors in Northeast Brazil. Int. J. Environ. Res. Public Health.

[8] Murr C., Widner B., Wirleitner B., Fuchs D. (2002). Neopterin as a marker for immune system activation. Curr. Drug Metabol..

[9] Michalak Ł., Bulska M., Strząbała K., Szcześniak P. (2017). Neopterin as a marker of cellular immunological response. Postep. Hig. I Med. Dosw..

[10] Hathaway-Schrader J.D., Novince C.M. (2021). Maintaining homeostatic control of periodontal bone tissue. Periodontology 2000.

[11] Omi M., Mishina Y. (2022). Roles of osteoclasts in alveolar bone remodeling. Genesis.

[12] Tassery H., Levallois B., Terrer E., Manton D.J., Otsuki M., Koubi S., Gugnani N., Panayotov I., Jacquot B., Cuisinier F. (2013). Use of new minimum intervention dentistry technologies in caries management. Aust. Dent. J..

[13] Gomez J. (2015). Detection and diagnosis of the early caries lesion. BMC Oral Health.

[14] D’Souza S., Addepalli V. (2018). Preventive measures in oral cancer: An overview. Biomed. Pharmacother..

[15] Komlós G., Csurgay K., Horváth F., Pelyhe L., Németh Z. (2021). Periodontitis as a risk for oral cancer: A case-control study. BMC Oral Health.

[16] Garg A., Chaturvedi P., Gupta P.C. (2014). A review of the systemic adverse effects of areca nut or betel nut. Indian J. Med. Paediatr. Oncol..

[17] Warnakulasuriya S., Chen T.H.H. (2022). Areca Nut and Oral Cancer: Evidence from Studies Conducted in Humans. J. Dent. Res..

[18] Trivedy C.R., Craig G., Warnakulasuriya S. (2002). The oral health consequences of chewing areca nut. Addict. Biol..

[19] Gibbins H.L., Proctor G.B., Yakubov G.E., Wilson S., Carpenter G.H. (2014). Concentration of salivary protective proteins within the bound oral mucosal pellicle. Oral Dis..

[20] Hannig C., Hannig M., Kensche A., Carpenter G. (2017). The mucosal pellicle—An underestimated factor in oral physiology. Arch. Oral Biol..

[21] Ployon S., Belloir C., Bonnotte A., Lherminier J., Canon F., Morzel M. (2016). The membrane-associated MUC1 improves adhesion of salivary MUC5B on buccal cells. Application to development of an in vitro cellular model of oral epithelium. Arch. Oral Biol..

[22] Alowais S.A., Alghamdi S.S., Alsuhebany N., Alqahtani T., Alshaya A.I., Almohareb S.N., Aldairem A., Alrashed M., Bin Saleh K., Badreldin H.A. (2023). Revolutionizing healthcare: The role of artificial intelligence in clinical practice. BMC Med. Educ..

[23] Hamet P., Tremblay J. (2017). Artificial intelligence in medicine. Metab. Clin. Exp..

[24] Nguyen T.T., Larrivée N., Lee A., Bilaniuk O., Durand R. (2021). Use of Artificial Intelligence in Dentistry: Current Clinical Trends and Research Advances. J. Can. Dent. Assoc..

